# The Stability of Ribosome Biogenesis Factor WBSCR22 Is Regulated by Interaction with TRMT112 via Ubiquitin-Proteasome Pathway

**DOI:** 10.1371/journal.pone.0133841

**Published:** 2015-07-27

**Authors:** Kadri Õunap, Lilian Leetsi, Maarja Matsoo, Reet Kurg

**Affiliations:** Institute of Technology, University of Tartu, Tartu, Estonia; Saint Louis University School of Medicine, UNITED STATES

## Abstract

The human WBSCR22 protein is a 18S rRNA methyltransferase involved in pre-rRNA processing and ribosome 40S subunit biogenesis. Recent studies have shown that the protein function in ribosome synthesis is independent of its enzymatic activity. In this work, we have studied the WBSCR22 protein interaction partners by SILAC-coupled co-immunoprecipitation assay and identified TRMT112 as the interaction partner of WBSCR22. Knock-down of TRMT112 expression decreased the WBSCR22 protein level in mammalian cells, suggesting that the stability of WBSCR22 is regulated through the interaction with TRMT112. The localization of the TRMT112 protein is determined by WBSCR22, and the WBSCR22-TRMT112 complex is localized in the cell nucleus. We provide evidence that the interaction between WBSCR22/Bud23 and TRMT112/Trm112 is conserved between mammals and yeast, suggesting that the function of TRMT112 as a co-activator of methyltransferases is evolutionarily conserved. Finally, we show that the transiently expressed WBSCR22 protein is ubiquitinated and degraded through the proteasome pathway, revealing the tight control of the WBSCR22 protein level in the cells.

## Introduction

Ribosome biogenesis is one of the most fundamental and energy consuming processes of the cell. The synthesis of ribosomes is a complex process involving several hundred genes functioning in transcription of precursor rRNAs, processing of pre-rRNAs, assembly of ribosomal proteins with pre-rRNAs, and nuclear export of the ribosomal particles. It requires several trans-acting factors at different stages along the pathway including more than 200 proteins that act to modify and cleave pre-rRNAs and help to assemble and export ribosomal particles [1]. These processes must be tightly regulated in space and time as defects in ribosome biogenesis may cause conditions known as ribosomopathies and increase suspectibility to cancer [2–4].

The WBSCR22 protein is a rRNA methyltransferase involved in pre-rRNA processing and ribosome 40S subunit biogenesis [5,6]. It affects the late steps in 18S rRNA processing as the depletion of WBSCR22 leads to the accumulation of 18S-E pre-rRNA intermediate in the cell nucleus [1,5,6]. The WBSCR22 protein mediates N^7^-methylation of G1639 in 18S rRNA, however, its catalytic activity is not required for 18S pre-rRNA processing [5,7]. WBSCR22 is the functional homologue of yeast Bud23 partially complementing the slow growth phenotype and ribosome synthesis defects of *bud23* deletion mutant [6]. Recent studies have shown that the WBSCR22 expression is upregulated in different cancers, including invasive breast cancer, multiple myeloma cells and hepatocellular carcinoma, and the cell growth is inhibited by treatment with WBSCR22 siRNA [6,8–10]. In addition, a loss of the WBSCR22 protein was observed in both inflammatory and neoplastic human lung pathologies, and in some cancer types [11]. WBSCR22 has been shown to modulate the histone methylation, specifically methylation of H3K79 and H3K9 in GILZ promoter [11] and H3K9 in Zac1 promoter region [9], however, the histone methyltransferase activity of WBSCR22 *in vitro* has not been shown.

The human TRMT112 protein is the homologue of *Saccharomyces cerevisiae*´s Trm112 (tRNA methyltransferase 11–2) which is a cofactor for different methyltransferases involved in tRNA, rRNA and protein methylation [12–17]. TRMT112 is an evolutionarily conserved protein which is highly and ubiquitously expressed in various organs and tissues during mouse embryonic development [18]. TRMT112 ortholog in *Arabidopsis thaliana*, Smo2 protein, functions in regulation of cell division progression during organ growth and disruption of SMO2 affects G2-M phase progression in cell cycle [19]. The human TRMT112 protein has been shown to interact with methyltransferases involved in translation, specifically with ALKBH8 (ABH8) and HEMK2α which methylate the wobble uridine (U34) of certain tRNAs and translation termination factor eRF1, respectively [20,21].

Recent studies have shown that the function of WBSCR22 in 40S subunit biogenesis is independent of its function as an RNA methyltransferase. The protein itself, rather than the enzymatic activity of WBSCR22 is required for small subunit synthesis. In this work, we have studied the interaction partners of WBSCR22 and identified TRMT112 as the major interacting protein of WBSCR22 in proliferating cells. We show that the stability of WBSCR22 in mammalian cells is regulated by the interaction with TRMT112. The localization of the TRMT112 protein is determined by WBSCR22, and the WBSCR22-TRMT112 complex is localized in the cell nucleus. We further report that the WBSCR22 protein is ubiquitinated and degraded through the 26S proteasome pathway. Our data suggest that the function of TRMT112 as a regulator or co-activator of methyltransferases is evolutionarily conserved.

## Results

### Analysis of WBSCR22 interaction partners

**Fig 1:**
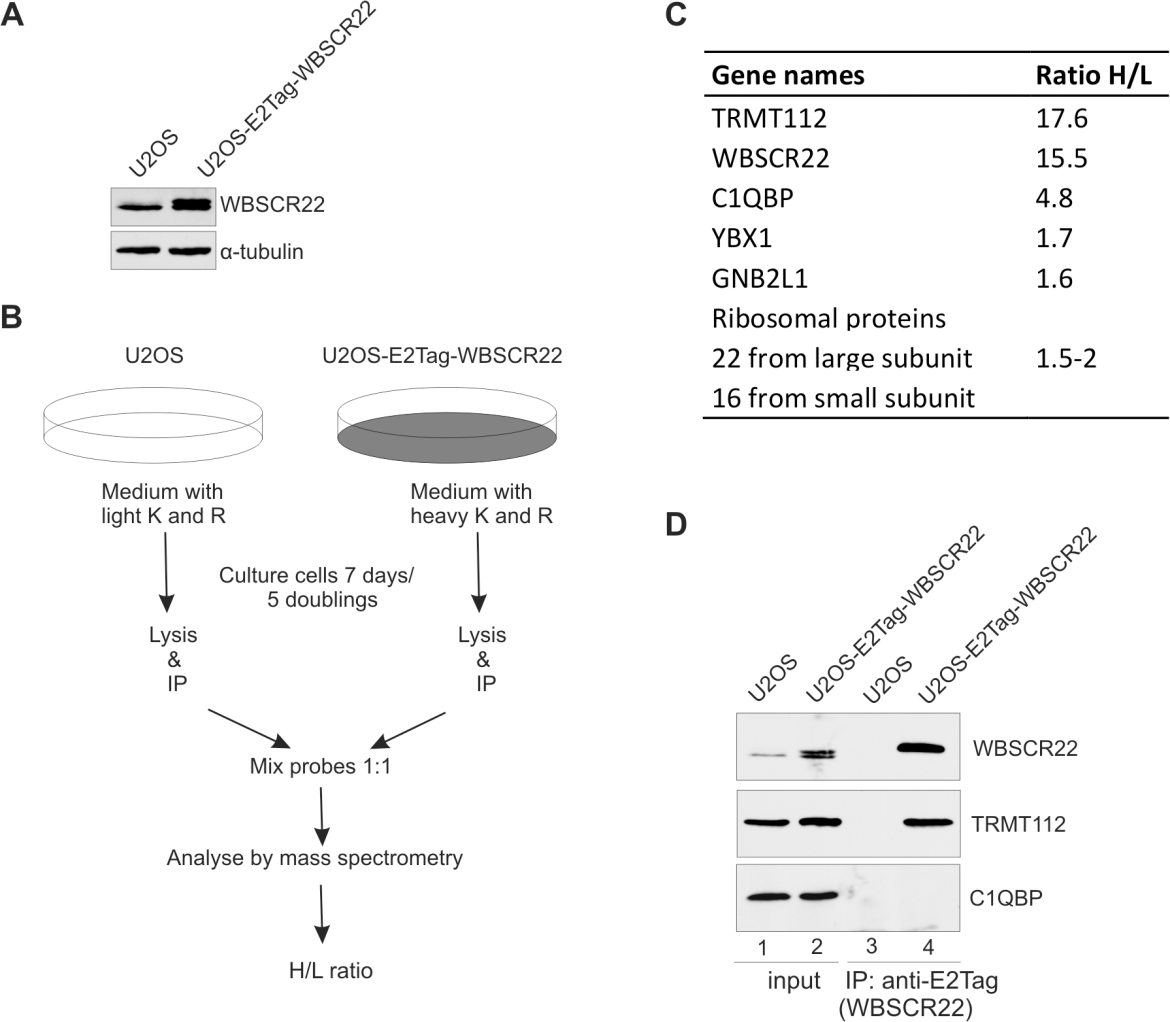
WBSCR22 interacts with the TRMT112 protein in mammalian cells. (A) WBSCR22 protein expression in U2OS and U2OS cell line stably expressing the E2Tag-WBSCR22 was determined by western blot using anti-WBSCR22 antibody. α-tubulin detected with anti-α-tubulin antibodies is used as a loading control. (B) Experimental scheme of the SILAC coupled co-immunoprecipitation assay performed. (C) The WBSCR22 protein interaction partners in U2OS cells stably expressing the WBSCR22 protein. H/L ratio shows the relative enrichment of the peptides of proteins pulled-down with WBSCR22 compared to mock control. (D) The WBSCR22 protein interacts with TRMT112. The WBSCR22 protein was immunoprecipitated from U2OS and U2OS-E2Tag-WBSCR22 cell lysates with an antibody against E2Tag. Proteins of the extract (Input; 10% of the lysate and pulled-down fraction (IP) were analyzed by immunoblotting with antibodies against WBSCR22, TRMT112 and C1QBP.

In order to investigate the proteins interacting with WBSCR22 in mammalian cells, we performed SILAC (stable isotope labeling by amino acids in cell culture) coupled co-immunoprecipitation assay. For this, we generated the U2OS cell line that stably expresses the WBSCR22 protein containing an epitope tag E2Tag in its N-terminus. As shown in Fig 1A, the expression level of recombinant WBSCR22 in stable cell line was comparable to that of the endogenous protein. The scheme of our SILAC experiment is shown in Fig 1B. We identified a total of 149 proteins from which 49 proteins had H/L ratio higher than 1.5 (S1 Table). Among the proteins with highest H/L ratio were WBSCR22 itself and TRMT112. The H/L ratio of TRMT112 was very similar to that of the WBSCR22 protein pulled-down from the cell lysates (Fig 1C). TRMT112 was also pulled-out in a similar study of Jangani et al. where HEK293T cells were transfected with Halo-tagged WBSCR22 [11]. TRMT112 is a homologue of *Saccharomyces cerevisiae´*s protein Trm112 that is known to interact with a variety of methyltransferases involved in different aspects of translation. Yeast Trm112 is also required for the activity of Bud23, the WBSCR22 yeast orthologue, in ribosome biogenesis [13,17]. We also identified several other proteins, including C1QBP and a number of ribosomal proteins from both small and large subunits as interaction partners of WBSCR22 in proliferating cells (Fig 1C). However, the H/L ratios of these proteins were lower compared to the TRMT112 protein.

In order to confirm the interactions identified by mass spectrometry, we performed the immunoprecipitation analysis followed by western blot. As shown in Fig 1D, the WBSCR22 protein was able to pull-down the TRMT112 protein, but not C1QBP1, from the U2OS cell lysates (Fig 1D, lane 4). C1QBP1 was originally described as a splicing factor ASF/SF2 interacting protein and is localized primarily to mitochondria [22]. The C1QBP1 protein has shown to interact with the plasma complement component C1q and with many different viral proteins [23]. At the same time it has also been described as a frequent contaminant in affinity purification/mass spectrometry approaches [24]. We could not confirm the interaction between WBSCR22 and C1QBP1 by co-immunoprecipitation, suggesting that they do not form a stable complex in the U2OS cells. The immunofluorescence analysis revealed that the TRMT112 protein is localized mainly to the nucleus and C1QBP1 to the cytoplasm in HeLa cells (S1 Fig). These data show that the TRMT112 protein is the main interaction partner of WBSCR22 in proliferating mammalian cells.

### WBSCR22 protein stability is regulated by TRMT112

**Fig 2:**
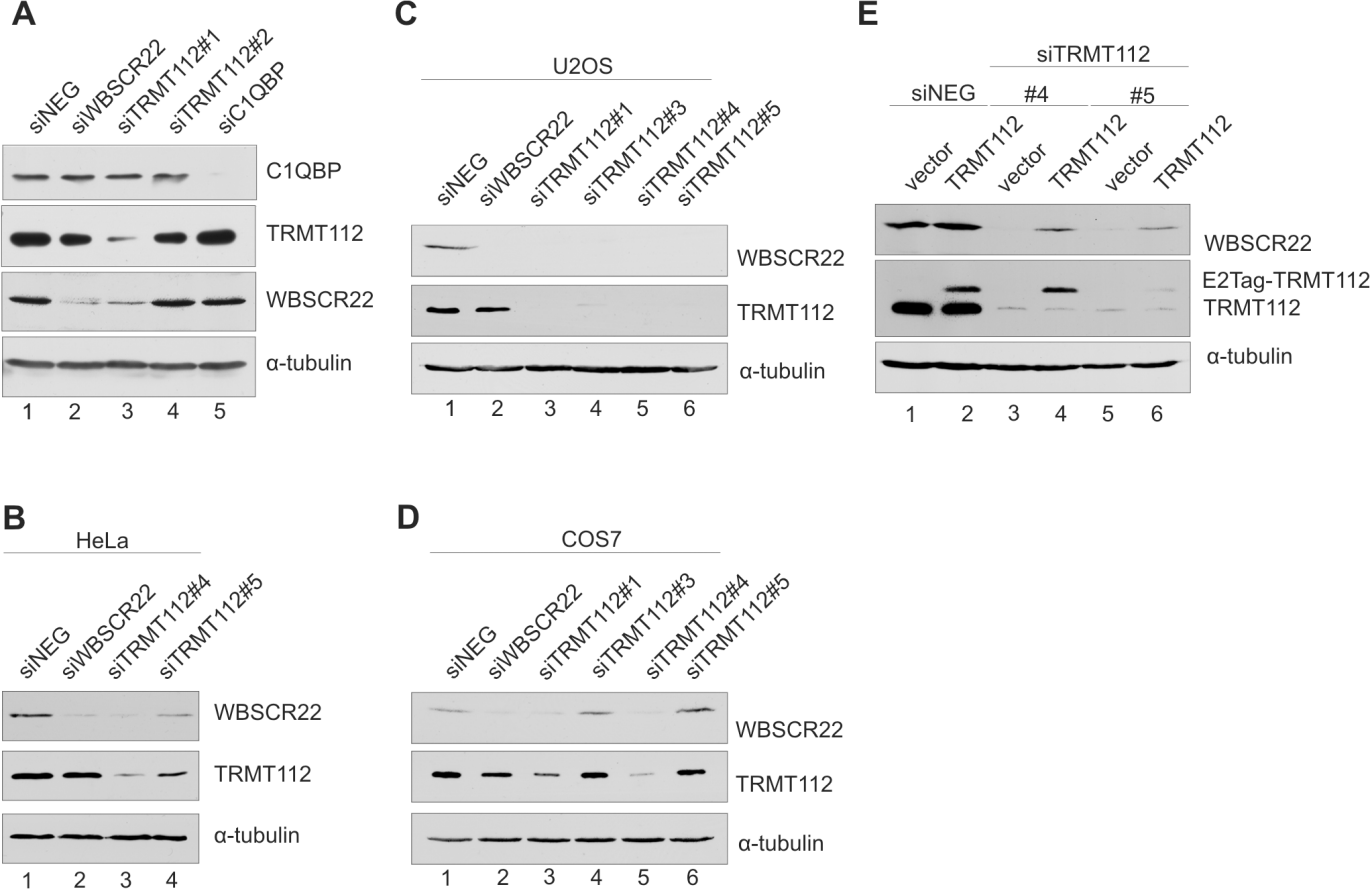
The stability of WBSCR22 protein is regulated by TRMT112. (A) The expression level of WBSCR22 and its interaction partners in response to siRNA treatment was analyzed by western blot in HeLa cells. The cells electroporated with siRNAs were collected 48 h after transfection, lysed and analyzed with antibodies against WBSCR22, TRMT112, C1QBP and α-tubulin. (B-D) TRMT112 is required for WBSCR22 stability in HeLa (B), U2OS (C) and COS-7 (D) cells. Cells were treated with TRMT112 siRNAs and analyzed by antibodies against WBSCR22, TRMT112 and α-tubulin. (E) Expression of TRMT112 restores the endogenous WBSCR22 protein level in TRMT112-depleted U2OS cells. First, the U2OS cells were electroporated with siRNAs and 48 hours later with the plasmid encoding for E2Tag-TRMT112. Cells were collected 24 hours after second transfection and analyzed by western blotting with antibodies against WBSCR22, TRMT112 and α-tubulin. Proteins from 10^5^ cells were loaded on each lane.

To study the functional significance of WBSCR22 interactions, we knocked down the expression of WBSCR22, C1QBP and TRMT112 with siRNAs in HeLa cells and analyzed the expression level of each of these proteins by immunoblotting. Treatment of cells with siTRMT112#1 reduced the expression level of the TRMT112 protein itself and caused a significant decrease of the WBSCR22 protein level in HeLa cells (Fig 2A, lane 3). At the same time another siRNA designed against TRMT112, siTRMT112#2, and the mock control, siNeg, did not have any effect on TRMT112 or WBSCR22 protein levels (Fig 2A, lane 4). Downregulation of C1QBP expression by RNA interference efficiently decreased the expression of the C1QBP1 protein, but did not affect the amount of WBSCR22 protein within the cells (Fig 2A, lane 5). Knock-down of WBSCR22 reduced the amount of the WBSCR22 protein itself, but did not affect the expression level of TRMT112 or C1QBP (Fig 2A, lane 2). To confirm the interaction between WBSCR22 and TRMT112 proteins within the cells, we used additional siRNAs to knock down the expression of TRMT112. Two siRNAs designed against the 3’UTR of TRMT112 both reduced the expression level of TRMT112 itself and the WBSCR22 protein in HeLa (Fig 2B) and U2OS (Fig 2C) cells. SiRNA knock-down was also performed in green monkey COS-7 cells used for immunoprecipitation experiments in this study. Both siRNAs #1 and #4 against TRMT112 efficiently reduced the TRMT112 as well as WBSCR22 protein levels in these cells (Fig 2D). TRMT112 siRNA #5 has three mismatches and siRNA#3 does not work in monkey COS-7 cells. In the rescue experiments, we knocked down the TRMT112 expression with siRNAs and transfected the cells with the pQM plasmid encoding for E2Tag-TRMT112. As shown in Fig 2E, exogenous expression of TRMT112 increased the endogenous WBSCR22 protein level in the siTRMT112-treated U2OS cells (lanes 4, 6). These data reveal that the TRMT112 protein affects the WBSCR22 protein level in all cell lines tested and suggests that TRMT112 is necessary for the stability of WBSCR22.

### The WBSCR22-TRMT112 complex is localized in the nucleus

**Fig 3:**
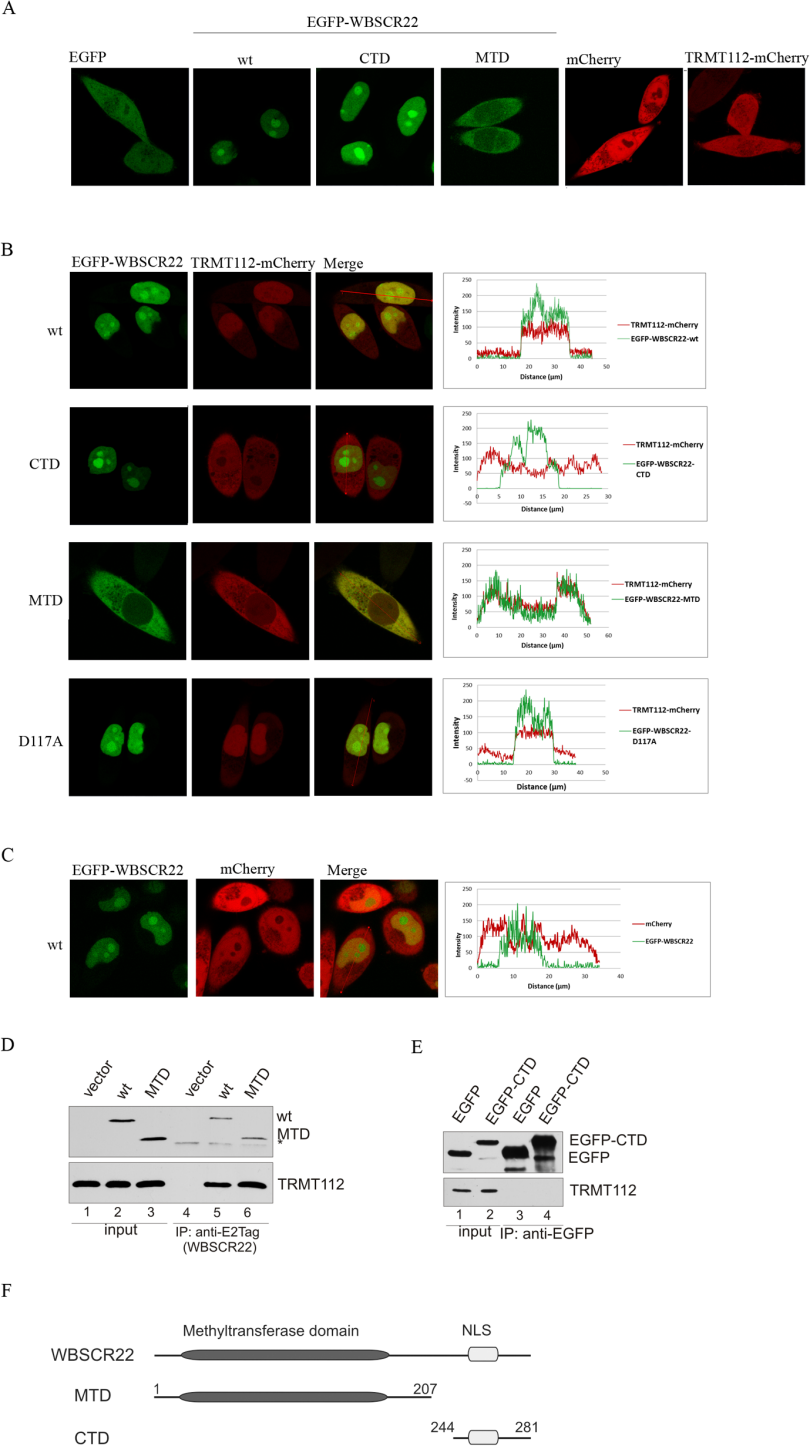
Localization of TRMT112 is determined by the WBSCR22 protein. (A) Live cell confocal images of EGFP, EGFP-WBSCR22, EGFP-WBSCR22-CTD, EGFP-WBSCR22-MTD, mCherry and TRMT112-mCherry proteins in HeLa cells. (B) Live cell confocal images and fluorescence intensity profile graphs of TRMT112-mCherry protein co-expressed with EGFP-WBSCR22, EGFP-WBSCR22-CTD, EGFP-WBSCR22-MTD and EGFP-WBSCR22-D117A, and (C) mCherry protein co-expressed with EGFP-WBSCR22. Fluorescence signal was visualized using confocal laser scanning microscope LSM710 (Zeiss). Images were obtained with 63x lens and analyzed by ZEN2011 software. (D) Co-immunoprecipitation of WBSCR22 and TRMT112 proteins. COS-7 cells were transfected with plasmids encoding for WBSCR22 and its mutant MTD. 24 hours later co-immunoprecipitation was performed using antibody against E2Tag. Immunoblotting was performed with antibodies against E2Tag (HRP-conjugate) and TRMT112. The non-specific signal is shown by asterisk. (E) Co-immunoprecipitation of EGFP-tagged WBSCR22-CTD protein. Immunoprecipitation was performed with magnetic beads that were covalently coupled with EGFP binding protein and analyzed by immunoblotting with antibodies against EGFP and TRMT112. (F) A schematic representation of WBSCR22 proteins.

Our next aim was to study the subcellular localization of the TRMT112 protein alone and together with WBSCR22 in living cells. For this, we fused the fluorescent protein EGFP to the N-terminus of the WBSCR22 and mCherry to the C-terminus of TRMT112. As shown in Fig 3A, both fluorescent proteins, EGFP and mCherry, were localized throughout the whole cell, to the nucleus and cytoplasm, when expressed transiently in HeLa cells. The TRMT112-mCherry protein was detected all over the cell, both in nucleus and cytoplasm (Fig 3A). The fluorescence signal of EGFP-WBSCR22 revealed that the WBSCR22 protein is localized in the nucleus and accumulated to the nucleolus (Fig 3A). The truncated WBSCR22 protein MTD, containing the methyltransferase domain of WBSCR22 (amino acids 1 to 207) and lacking the C-terminal domain (CTD) containing the nuclear localizing signal (NLS) (Fig 3F), was detected in the cytoplasm, and the CTD (38 amino acids from C-terminus) localized to the nucleus and nucleoli of the cell (Fig 3A).

**Fig 4:**
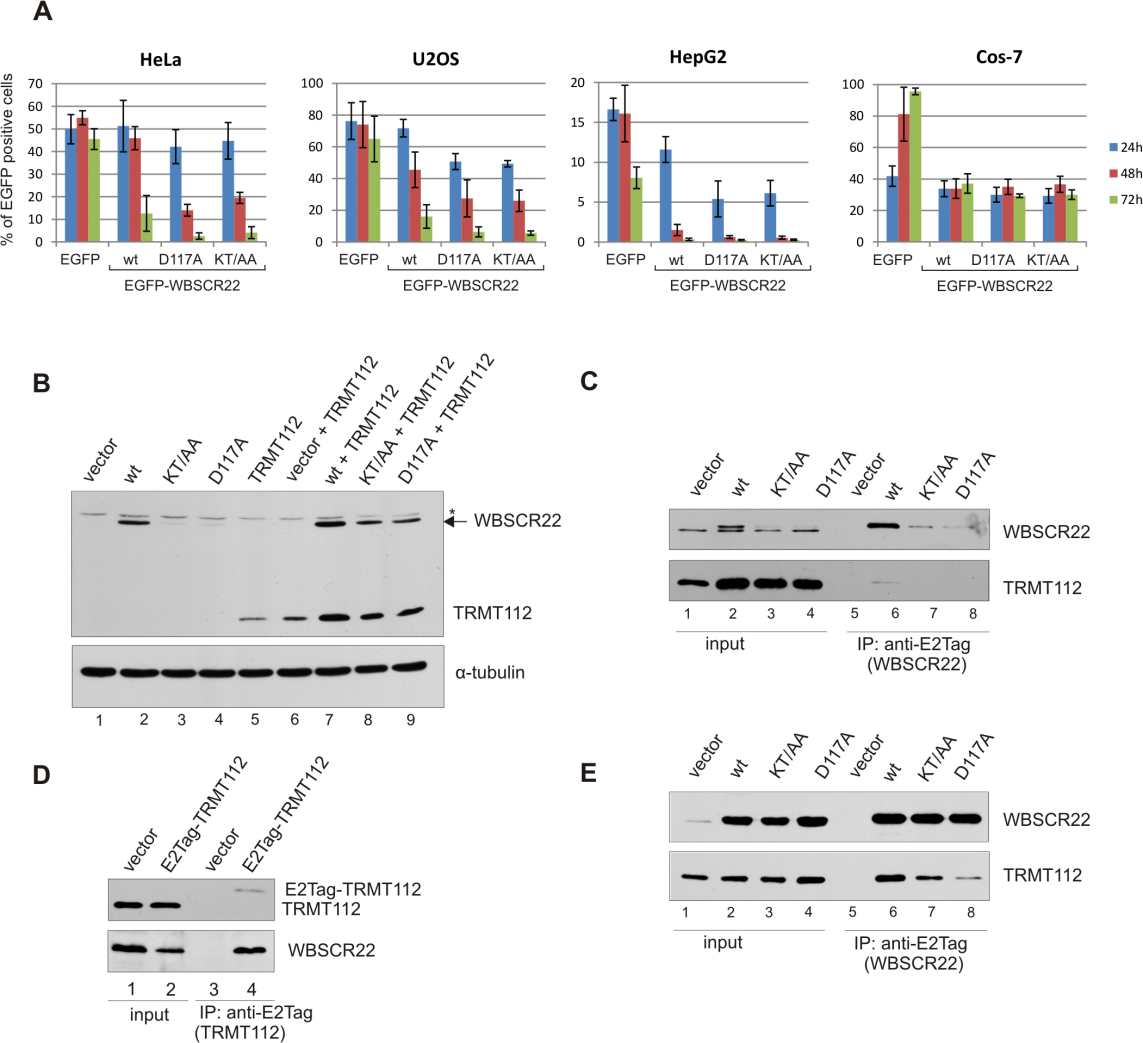
Expression of WBSCR22 mutants defective in TRMT112 binding. (A) Hela, U2OS, HepG2 and COS-7 cells were transfected with plasmids expressing EGFP, EGFP-WBSCR22, EGFP-WBSCR22-KT/AA and EGFP-WBSCR22-D117A proteins. The amount of EGFP positive cells was analyzed by flow cytometry 24, 48 and 72 hours after transfection. (B) Expression of TRMT112 and WBSCR22 proteins in HeLa cells. HeLa cells were transfected with plasmids encoding for wt WBSCR22 and its mutants with and without plasmid for TRMT112. Transfected cells were harvested 24 hours after electroporation, the lysate of 10^5^ cells was loaded on each lane and analyzed by western blot using antibody against E2Tag and α-tubulin. The non-specific signal is shown by asterisk. (C-E) Co-immunoprecipitation of WBSCR22 and TRMT112 proteins. HeLa (C) and COS-7 (E) cells were transfected with plasmids encoding for wt and mutant WBSCR22 proteins and lysed 24 hours later. (D) HeLa cells were transfected with plasmid encoding for E2Tag-TRMT112. In all cases, co-immunoprecipitation was performed using antibody against E2Tag and immunoblotting with antibodies against WBSCR22 and TRMT112. Proteins of the extract (Input; 10% of cell lysate) and pulled-down fraction (IP) were analyzed by immunoblotting.

In order to follow the localization of WBSCR22-TRMT112 complex, we expressed the EGFP-WBSCR22 and TRMT112-mCherry proteins together. As shown in Fig 3B, the fluorescence signals of both proteins were detected in the nucleus of the cell. The WBSCR22-TRMT112 complex was mostly detected in the nucleoplasm, but also in the nucleolus of the cell. Interestingly, we did not detect accumulation of WBSCR22-TRMT112 complex to the nucleolus typical for WBSCR22 protein alone (Fig 3B). In case of co-expression of EGFP-WBSCR22 and mCherry, the red fluorescence signal was detected both in cytoplasm and nucleus, but was excluded from the nucleolus (Fig 3C). This confirms that re-localization of TRMT112 protein to the nucleus is determined by WBSCR22, and that TRMT112-mCherry signal in the nucleus and nucleolus is specific to WBSCR22-TRMT112 complex (Fig 3C). The localization pattern of co-expressed TRMT112 and WBSCR22 is also shown by fluorescence intensity profiles (Fig 3B, 3C; right panels). Co-expression of CTD and TRMT112 revealed that CTD localized similarly to WBSCR22, to the nucleus and nucleoli of the cell, while the TRMT112 protein localization was detected throughout the whole cell, in the nucleus as well as in cytoplasm (Fig 3B). So, the expression of CTD did not affect the subcellular localization of TRMT112 as the fluorescence intensity of TRMT112 was similar in the nucleus and cytoplasm of the cell. Analysis of MTD and TRMT112 revealed a similar pattern of cytoplasmic localization for both proteins (Fig 3B), suggesting that TRMT112 interacts with MTD in the cytoplasm of the cell. The WBSCR22 mutant D117A, which has a reduced binding to TRMT112 (Fig 4D), also localized TRMT112 to the nucleus, but some protein remained in the cytoplasm (compare intensity profiles of Fig 3B). To rule out the possibility that TRMT112-mCherry forms a complex with endogenous WBSCR22 in D117A expressing cells, we repeated this experiment with WBSCR22-depleted cells. The WBSCR22 mutant D117A localized TRMT112-mCherry both in the cytoplasm and nucleus of the cell after knockdown of endogenous WBSCR22 (S2 Fig). So, the depletion of WBSCR22 decreased the amount of nuclear D117A-TRMT112 complex suggesting that the endogenous WBSCR22 masked the effect of D117A mutant by forming a complex with the over-expressed TRMT112 protein.

To confirm the interaction between TRMT112 and the methyltransferase domain of WBSCR22, we used co-immunoprecipitation followed by immunoblot analysis. WBSCR22 and MTD were expressed as E2Tag-fused proteins and affinity purified from cell lysates using the antibody against E2Tag. Both proteins were able to pull-down TRMT112 (Fig 3D), suggesting that the methyltransferase domain is necessary and sufficient for the interaction with TRMT112. The CTD fused with EGFP was not able to co-immunoprecipitate TRMT112 from cell lysates (Fig 3E). Taken together, these results show that the WBSCR22-TRMT112 complex is localized to the cell nucleus including nucleolus, and the subcellular localization of the complex is determined by the WBSCR22 protein.

### Expression of WBSCR22 mutants defective in TRMT112 binding

In order to analyze the biological function of WBSCR22-TRMT112 interaction in more detail, we constructed WBSCR22 mutants with point-mutations in the interface of WBSCR22-TRMT112 complex. The crystal structure of yeast Bud23-Trm112 complex reveales that the interaction interface is characterized by the presence of a central large hydrophobic core surrounded by polar residues involved in the formation of hydrogen bonds and salt bridges [25]. A sequence alignment of WBSCR22 and yeast Bud23 shows that most of the amino acids involved in complex formation are similar or identical in these two proteins [25]. Based on the conservation, we generated two mutants, first WBSCR22-D117A, where the conserved Asp117 is replaced with alanine, and second, the double-mutant WBSCR22-KT/AA, where amino acids K112 and T115 are both mutated to alanines. The amino acid D112 in Bud23, corresponding to D117 in WBSCR22, is involved in the formation of salt bridges in the interface of Bud23-Trm112 complex, and resulted in strongly impaired Bud23 stability in yeast [17,25]. K112 and T115 of WBSCR22 are engaged in formation of hydrogen bonds and are not identical between yeast and human. All the mutated WBSCR22 proteins contain an epitope tag E2Tag in their amino-terminus.

To follow the expression of EGFP-WBSCR22 proteins in mammalian cells, we first performed the flow cytometry analysis. HeLa cells were transfected with plasmids encoding for wt WBSCR22 and its mutants, and the number of EGFP positive cells was measured 24, 48 and 72 hours after electroporation. As shown in Fig 4A, 24 hours after transfection approximately 50% of the cells express the EGFP and EGFP-WBSCR22 proteins and about 40% of the cells express mutant proteins EGFP-WBSCR22-D117A and EGFP-WBSCR22-KT/AA in HeLa cells. The expression of recombinant proteins from transient expression vectors, which do not replicate within the cells, usually achieves a maximum 48 hours after transfection. This was also seen in our experiment with EGFP and EGFP-WBSCR22 proteins. However, the number of cells expressing mutant proteins EGFP-WBSCR22-D117A and EGFP-WBSCR22-KT/AA was reduced to 15–20% at 48 hours post-transfection. Three days after transfection, the amount of cells that express the wt EGFP-WBSCR22 protein was decreased to 13% and we were not able to detect the cells expressing EGFP-WBSCR22 mutant proteins. The expression level of control protein EGFP did not change during this time (Fig 4A). Similar expression pattern was observed in other human cell lines, in osteosarcoma cells U2OS and hepatocellular carcinoma cells HepG2 (Fig 4A).

Next we transfected the HeLa cells with plasmids encoding for WBSCR22 wt, KT/AA and D117A proteins and analyzed their expression by western blotting using anti-E2Tag antibodies. As shown in Fig 4B, the WBSCR22 mutant proteins KT/AA and D117A had a reduced expression level compared to wt WBSCR22 (lanes 2–4). Co-expression with TRMT112, containing E2Tag in its C-terminus, enhanced the expression level of wt WBSCR22 as well as mutant proteins (Fig 4B, lanes 7–9). However, the expression level of D117A and KT/AA remained lower than that of wt WBSCR22. The expression of TRMT112 was also enhanced when co-expressed with WBSCR22 (lanes 6,7), showing that WBSCR22 and TRMT112 form a stable complex and stabilize each other within the cells.

In order to study the effect of WBSCR22 point-mutations on WBSCR22-TRMT112 interaction, we first performed the immunoprecipitation assay in HeLa cells. We transfected the HeLa cells with plasmids encoding for wt or mutated WBSCR22 proteins and performed the co-immunoprecipitation analysis with an antibody against E2Tag. The wt WBSCR22 protein was able to pull-down the endogenous TRMT112 from cell lysates (Fig 4C) and E2Tag-TRMT112 in turn co-immunoprecipitated the endogenous WBSCR22 protein (Fig 4D). We also tried to immunoprecipitate the WBSCR22 mutant proteins KT/AA and D117A from HeLa cells, but their expression level was too low to perform immunoprecipitation assay. To achieve the higher expression level of mutant WBSCR22 proteins suitable for co-immunoprecipitation analysis, we have used the green monkey kidney COS-7 cells. The expression plasmids used in our work contain the SV40 origin and are able to replicate in COS-7 cells expressing the SV40 large T-antigen. This guarantees the high expression level of recombinant proteins for several days as shown by FACS (Fig 4A) and western blot (Fig 4E; input) analysis. Similar to HeLa and U2OS cells, the wt WBSCR22 protein was able to co-immunoprecipitate the endogenous TRMT112 protein from the COS-7 cell lysates. WBSCR22 mutant proteins D117A and KT/AA co-immunoprecipitated less TRMT112 than wt WBSCR22 (Fig 4E, lanes 7–8). Mutation of Lys112 and Thr115 to alanines resulted in slightly reduced TRMT112 binding, and mutation of highly conserved Asp117 to alanine had a severly reduced activity in this assay. These results suggest that all these amino acids are involved in forming the interaction surface between WBSCR22 and TRMT112. However, both mutations reduced rather than abolished the interaction between WBSCR22 and TRMT112.

### The TRMT112 binding activity and nuclear localization of WBSCR22 is required for its activity in yeast

**Fig 5:**
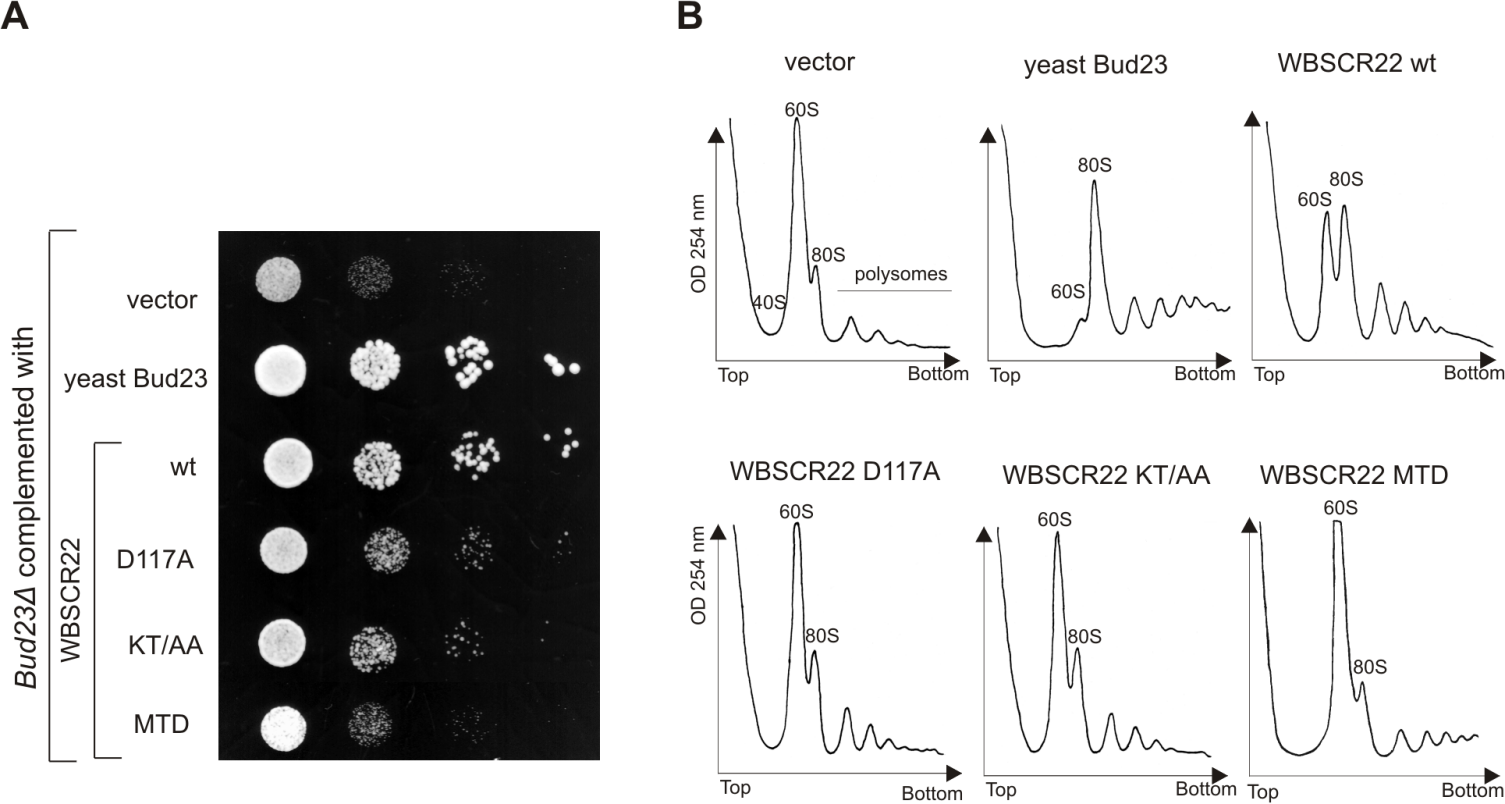
WBSCR22 interaction with TRMT112 is conserved in mammals and yeast. Analysis of *bud23Δ* yeast strain complemented with human WBSCR22, its point mutants WBSCR22-D117A and WBSCR22-KT/AA and MTD containing WBSCR22 methyltransferase domain. (A) For growth dilution assays, cultures were grown overnight and diluted to final optical density at OD_600_ 0.1, from which further 10-fold serial dilutions were spotted. Plates were incubated at 30°C for 3 days. (B) Polysome profiles of *bud23Δ* carrying plasmids for Bud23, WBSCR22, WBSCR22-D117A, WBSCR22-KT/AA, WBSCR22-MTD or vector as a control. Cell lysates were centrifuged on 10–45% sucrose gradient. Absorbance at 254 nm was measured across the gradient, and the positions corresponding to the 40S, 60S and 80S ribosomal particles are indicated.

We have previously shown that the WBSCR22 protein is the functional homologue of Bud23 partially complementing the slow growth and ribosome biogenesis defects of yeast *bud23∆* strain [6]. Next we analyzed the functional activity of WBSCR22 mutants defective in TRMT112 binding in yeast system. For this, the coding sequences of WBSCR22-KT/AA and WBSCR22-D117A mutants were cloned into yeast expression vector and their ability to complement the slow growth and ribosome biogenesis defects in yeast *bud23∆* strain was tested. As shown in Fig 5A, WBSCR22 complemented the slow growth of *bud23∆* strain partially as shown before [6]. WBSCR22 mutants KT/AA and D117A complemented the slow growth phenotype of *bud23∆* strain to a much lesser extent than wt WBSCR22 (Fig 5A). However, KT/AA and D117A-complemented *bud23∆* strain grew better than negative control which was *bud23∆* strain complemented with vector. Similar results were obtained when we analyzed the ribosome profiles of complemented cells by sucrose gradient centrifugation (Fig 5B). The *bud23∆* strain exhibits a strong 40S biogenesis defect resulting in subunit imbalance with almost no free 40S, very high free 60S levels and reduced amount of assembled 80S ribosomes and polysomes [26]. The expression of yeast Bud23 protein suppressed the accumulation of free 60S and enhanced the formation of mature 80S particles and polysomes. The wt WBSCR22 complemented the defects of 40S ribosomes assembly partially, and WBSCR22 mutants defective in TRMT112 binding, KT/AA and D117A, had reduced activity in this assay compared to wt WBSCR22. These data suggest that the interaction with Trm112 is necessary for the WBSCR22 activity in yeast, as mutations on the WBSCR22-TRMT112 interaction surface resulted in the decrease of WBSCR22 activity. The truncated WBSCR22 protein MTD did not support the growth of *bud23∆* strain nor complement the defects of 40S ribosomes assembly (Fig 5A, 5B). MTD contains the methyltransferase domain of WBSC22, but lacks the C-terminal CTD required for nuclear/nucleolar localization of WBSCR22 (Fig 3A). These results show that the nuclear localization of WBSCR22 is also necessary for the WBSCR22 activity in yeast.

### The WBSCR22 protein level is regulated by ubiquitin-proteasome pathway

**Fig 6:**
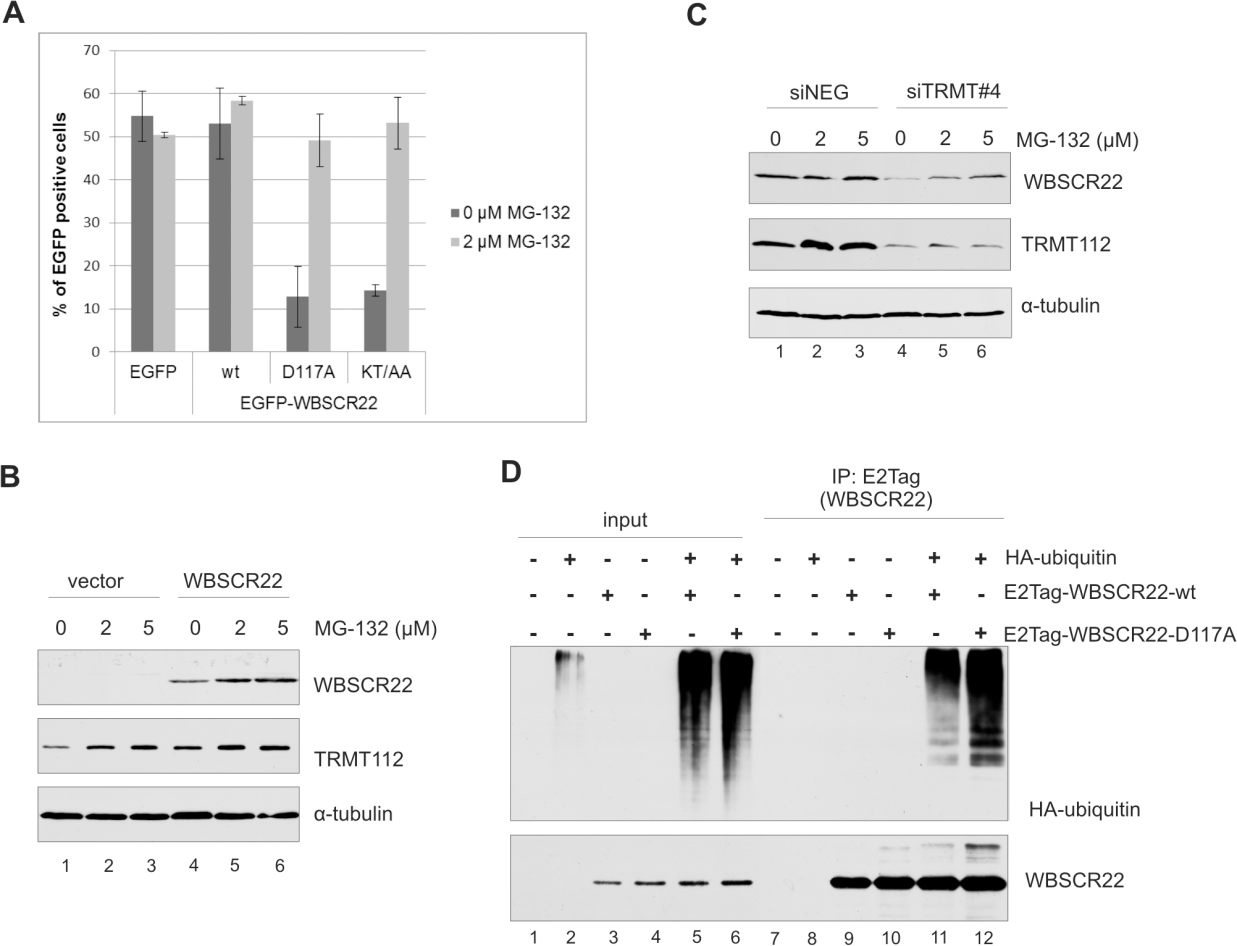
The WBSCR22 protein level is regulated by ubiquitin-proteasome pathway. (A) Flow cytometric analysis of HeLa cells transfected with plasmids encoding for EGFP, EGFP-WBSCR22, EGFP-WBSCR22-KT/AA and EGFP-WBSCR22-D117A proteins treated with proteasome inhibitor MG132. 24 hours after transfection cells were treated with MG132 for 16 hours, cells were harvested and the amount of EGFP positive cells was analyzed by flow cytometry. (B) Analysis of HeLa cells expressing the WBSCR22 protein treated with MG132 by immunoblotting. For western blot analysis, antibodies against E2Tag, TRMT112 and tubulin were used. (C) Western blot analysis of HeLa cells treated with TRMT112 siRNA and MG132. HeLa cells were transfected with siRNA and MG132 was added 56 hours later. Cells were collected 72 h post-transfection, and analyzed by immunoblotting with antibodies against WBSCR22, TRMT112 and tubulin. (D) WBSCR22 is ubiquitinylated in the cells. HeLa cells were transfected with plasmids encoding E2Tag-WBSCR22, E2Tag-WBSCR22-D117A and HA-Tag-ubiquitin and 24 hours later treated with 10 μM MG132 for 4 hours. Co-immunoprecipitation was performed with anti-E2Tag antibody, the samples were analyzed by immunoblotting using antibodies against WBSCR22 and HA-tag. Proteins of the extract (Input; 3%) and pulled-down fraction (IP) were analyzed by immunoblotting.

The WBSCR22 and TRMT112 proteins form a stable complex in the cells and the stability of WBSCR22 is regulated by TRMT112. Our previous experiments have also shown that the WBSCR22 expression level is controlled in the cells (Fig 4A). The concentration of particular proteins and degradation of misfolded proteins is often regulated by proteasomes. To investigate whether the WBSCR22 protein level in the cell is regulated by ubiquitin-proteasome degradation pathway, we examined the number of EGFP-WBSCR22 expressing cells in the presence or absence of the cell-permeable proteasome inhibitor MG132. MG132 is a peptide aldehyde, which effectively blocks the proteolytic activity of 26S proteasome complex with IC50 of approximately 5 μM for 24 h in HeLa cells [27]. HeLa cells were transfected with plasmids encoding for EGFP, EGFP-WBSCR22 and its mutants, 24 hours post-transfection the proteasome inhibitor MG132 was added and the cells were analyzed 16 hours later by flow cytometry. As shown in Fig 6A, treatment with MG132 slightly increased the amount of EGFP-WBSCR22-expressing cells but did not affect the number of EGFP expressing cells. The expression of mutant proteins EGFP-WBSCR22-D117A and EGFP-WBSCR22-KT/AA increased from 13% to 50% after treatment with MG132 (Fig 6A). To confirm that the WBSCR22 protein expression is regulated by proteasomes, we performed western blot analysis. As shown in Fig 6B, treatment with MG132 increased the expression level of the WBSCR22 protein in HeLa cells (lanes 4–6). MG132 treatment also slightly increased the endogenous TRMT112 protein level, but did not affect the amount of tubulin (Fig 6B). This may refer that the level of TRMT112 is also controlled by proteasomes in the cell. In a different experiment, we knocked down the expression of TRMT112 with siRNA and treated the cells with MG132. As shown in Fig 6C, inhibition of proteasomes increased the level of endogenous WBSCR22 protein in TRMT112-depleted cells (lanes 4–6). These data suggest that the WBSCR22 protein, which is not in complex with TRMT112 or is not able to form a complex, is unstable and degraded by proteasomes.

Next we determined whether the WBSCR22 protein is ubiquitinated in the cells. For that, HeLa cells were co-transfected with HA-tagged ubiquitin and E2Tag-WBSCR22, and WBSCR22 was immunoprecipitated with antibody against E2Tag. Western blot analysis using antibody against HA-tag showed high-molecular weight bands of poly-ubiquitinated molecules in WBSCR22 pull-down fractions (Fig 6D, lane 11). HA tagged ubiquitin was specifically co-immunoprecipitated with E2-tagged WBSCR22 since the HA-ubiquitin was not immunoprecipitated from cells transfected with HA-ubiquitin alone (Fig 6D, lane 8). The poly-ubiquitin chains were also added to the WBSCR22 mutant D117A defective in TRMT112 binding (Fig 6D, lane 12). Taken together, these results suggest that the WBSCR22 is ubiquitinated and its expression level is controlled by the 26S proteasome-dependent proteolysis.

## Discussion

In this study, we investigated the interaction partners of WBSCR22 in mammalian cells and pulled-down TRMT112 as the major interaction protein of WBSCR22. We show that the WBSCR22-TRMT112 interaction modulates the stability of the WBSCR22 protein in the cells. This conclusion is supported by two different experiments. First, the WBSCR22 protein level decreased in response to TRMT112 siRNA treatment of the cells. Second, WBSCR22 mutants defective in TRMT112 binding disappeared from the cells faster than wild-type WBSCR22. These data suggest that the WBSCR22 protein level in the cell is under tight control and is regulated by the interaction with TRMT112. Previously yeast Trm112 was shown to interact with and stabilize Bud23, the yeast WBSCR22 homologue [13,17]. Our work suggests that the function of TRMT112/Trm112 as a regulator of methyltransferases and their activity is evolutionarily conserved.

Our data show that the stability of WBSCR22 is modulated by the interaction with the TRMT112 protein. We suggest that the amount of the WBSCR22 protein in the cells is regulated by TRMT112 molecules available for the interaction with WBSCR22, and that this is one mechanism to control the amount of the functionally active WBSCR22 protein in the cells. The TRMT112 protein has probably many different interaction partners in the cell similar to its yeast counterpart, and they compete with each other for TRMT112. We also show that the transiently expressed WBSCR22 protein is poly-ubiquitinated in the cells and its expression level is controlled by the 26S proteasome-dependent proteolysis. Protein degradation is important for the maintenance of protein homeostasis in cells. This is one mechanism to switch off the protein function in the cell and get rid of misfolded proteins, about 80% of intracellular protein breakdown occurs via the ubiquitin-proteasome system [28,29]. It is possible that the TRMT112 molecule functions as a chaperone and assists the proper folding of newly synthesized WBSCR22. Analysis of the WBSCR22 protein interactome in transiently transfected HEK293 cells identified ubiquitin and ubiquitination enzymes as interacting partners of WBSCR22 [11]. At the same time we did not identify any proteins linked to ubiquitination pathway in our SILAC screen with U2OS cells stably expressing the WBSCR22 protein (Fig 1). So, the ubiquitin-proteasome system may be triggered in response to high expression level of WBSCR22. However, we cannot rule out the possibility that the WBSCR22 protein has different partners in the cells of diverse origin, and that the factors which determine the abundance of the protein vary between cell types and different cell lines.

We provide evidence that the WBSCR22-TRMT112 complex is localized in the cell nucleus and that this is determined by the WBSCR22 protein. TRMT112 is a small protein which is detected in the nucleus and cytoplasm of the cell with no preference to any compartment when expressed alone. This is consistent with *in silico* predictions that about 51% of the protein is localized in the cytoplasm [30]. Our data suggest that TRMT112 binds to the methyltransferase domain of WBSCR22 in the cytoplasm and is then transported to the nucleus. Support for this notion comes from our experiments with truncated protein MTD, which is able to bind TRMT112 and is predominantly localized to the cytoplasm (Fig 3). However, the WBSCR22 mutant D117A, defective in TRMT112 binding, also localized TRMT112 into the cell nucleus to some extent. Knockdown of endogenous WBSCR22 reduced the amount of TRMT112-mCherry and D117A complex in the nucleus suggesting that over-expressed TRMT112 forms a complex with the endogenous WBSCR22 protein. Besides, it is possible that some other activities, like methyltransferase activity or interactions with other proteins, are also required for the localization of WBSCR22-TRMT112 complex in the cell nucleus. On the other hand, the mutation in position D117A does not disrupt the interaction between WBSCR22 and TRMT112 completely, therefore we cannot rule out the possibility that the two proteins may still interact at higher concentrations.

The WBSCR22-TRMT112 complex localizes all over the nucleus and does not accumulate to the nucleolus while the free WBSCR22 protein does. First, it is possible that all the WBSCR22 protein expressed in the cell is not in complex with TRMT112, and the free WBSCR22 still accumulates to the nucleolus. Second, this may refer that only limited amount of WBSCR22-TRMT112 complexes can associate with the nucleolus. The third possibility is that WBSCR22 and TRMT112 are in dynamic, rather than stable complex and that only the free WBSCR22 protein is associated with its putative target, pre-ribosomes or rRNA. We favour the model that WBSCR22 associates with pre-ribosomes transiently, because all our efforts to show the stable association with WBSCR22 and pre-ribosomes have failed [6]. This is in contrast with Bud23 which has been shown to co-sediment with 90S pre-ribosomal subunits, thus stably associate with them [13]. Furthermore, it is possible that the WBSCR22-TRMT112 complex has different tasks in nucleoplasm and nucleolus; it regulates the level of functionally active WBSCR22 protein in the nucleoplasm, and is required for pre-rRNA processing occurring presumably in the nucleolus of the cell.

The WBSCR22 is localized in the nucleus and accumulated to the nucleolus of the cell analyzed by live cell microscopy. Similarly to WBSCR22, its orthologues in yeast and *Arabidopsis thaliana*, Bud23 and Rid2, respectively, localize in the nucleoli when fused with green fluorescent protein and over-expressed in the cells [26,31]. At the same time the endogenous WBSCR22 protein is detected throughout the whole nucleus with no obvious accumulation to the nucleoli [6]. This supports our model that the amount of the protein required for its activity in the nucleolus is relatively low and/or is tightly regulated. The localization of over-expressed and endogenous TRMT112 proteins also differ from each other, the recombinant TRMT112 is localized both in cytoplasm and nucleus, while the endogenous protein is detected mainly in the nucleus (S1 Fig) [7]. We favour the explanation that all the TRMT112 protein is in complex with methyltransferases, and these interactions determine its final localization in the cells. In case of over-expression, there are not enough protein partners for TRMT112, and the protein is localized all over the cell. Interestingly, the WBSCR22-TRMT112 complex is detected throughout the whole nucleus including the nucleolus, while the endogenous TRMT112 is excluded from this compartment (S1 Fig) [7]. This may explain why only WBSCR22 is enriched relative to TRMT112 in the nucleolus in our live cell microscopy experiments.

We show that the interaction between WBSCR22 and TRMT112 is conserved between mammals and yeast. This is consistent with the work of Zorbas et al. [7]. Recently two different groups showed that TRMT112 homologue in yeast, Trm112, is required for Bud23 activity in ribosome synthesis [13,17]. Trm112 is a small protein which functions as a co-activator modulating the methyltransferase activity of at least four methyltransferases in yeast. Probably the structural elements involved in Trm112 interaction with yeast RNA Mtases are conserved enabling competetive interactions with at least four different methyltransferases (Bud23, Mtq2, Trm9 and Trm11) simultaneously. Our results show that the functional interaction between yeast Trm112 and human WBSCR22 occurs within the cells as WBSCR22 mutants defective in TRMT112 interaction did not complement the growth of *bud23* deletion strain as effifiently as the wt protein (Fig 5). This suggests that the overall structure and interaction surface of WBSCR22/Bud23 with TRMT112/Trm112 is conserved, in spite of the low conservation of TRMT112 proteins on amino acid level. In addition to WBSCR22, the human TRMT112 interacts with another methyltransferase, ALKBH8 in mammalian cells [21,32]. ALKBH8 homologue in yeast is Trm9 which is also a partner of Trm112 [15]. Comparison of the sequence of potential TRMT112 binding site of WBSCR22 with that of the ALKBH8 revealed that they are highly similar. This suggests that the structure of TRMT112/Trm112, and function as a regulator of methyltransferases and their activity is evolutionarily conserved.

The WBSCR22 protein is involved in rRNA processing and ribosome 40S subunit biogenesis [6]. It is required for late nuclear pre-ribosomal RNA processing as the down-regulation of WBSCR22 results in impaired 18S rRNA maturation [5,7]. Its role in rRNA processing supports the idea of WBSCR22 as a component of the ribosome biogenesis quality control mechanism. Ribosome synthesis is an energy consuming task which must be finely tuned to adopt the needs of the cell. However, cancer cells demand a global increase in protein synthesis to support their hyper-proliferative behaviour. It is possible that small discrepancies in quality control system may lead to defects in ribosome synthesis and translation. In the present work we show that the stability of WBSCR22 in the cells is modulated by the interaction with the TRMT112 protein. Furthermore, the level of the WBSCR22 protein, involved in regulation of rRNA processing and ribosome biosynthesis, is itself controlled by the cellular control system, the ubiquitin-proteasome pathway. Future work is now needed to establish the mechanisms and importance of these findings to ribosome biogenesis and cell proliferation.

## Materials and Methods

### Plasmids

WBSCR22 expression plasmid pQM-NTag-WB22 and yeast vectors pRS315-Bud23 and pRS315-WBSCR22 have been described previously [6]. WBSCR22 point-mutation were introduced by primer-directed mutagenesis using primers WB_K112T115AA_F (for WBSCR22-KT/AA) and WB_D117A_F (for WBSCR22-D117A) (all primers used in this study are listed in S2 Table). For pQMHSP-WBSCR22, XbaI and BglII fragment from pQM-NTag-WB22 was cloned into pQM-HSP-N/A. Primers pCGseq2 and WB22del2R were used to amplify N-terminus from pQM-NTag-WB22, product was cloned into BglII and HindIII sites of pQM-CMV-E2-N/A (Icosagen), yielding pQM-WBSCR22-MTD. For EGFP expression vectors, primers WB22-BglF and WB22-KpnR2 were used to amplify WBSCR22 coding sequences from pQM vectors. PCR products were cloned into the BglII and KpnI sites of pEGFP-C1 (Clontech) yielding pEGFP-WBSCR22, pEGFP-WBSCR22-MTD, pEGFP-WBSCR22-KT/AA and pEGFP-WBSCR22-D117A. Primers WB22_aa244 and pCG_AS were used to amplify WBSCR22 C-terminus from pQM-NTag-WB22. PCR product was cloned into HindIII site of pEGFP-C1 yielding pEGFP-WBSCR22-CTD. mCherry coding sequence was amplified by primers CherC-F and CherC-R and cloned into Acc65I and BglII sites of pQM-C/A vector (Icosagen) resulting in pQM-CTag-mCherry. Human TRMT112 cDNA was amplified from HeLa cells by PCR using primers TRMTBgl-F and TRMT-R. The product was cut with BglII and Acc65I and cloned into the BamHI and Acc65I sites of pQM-CTag-mCherry or pQM-C/A, yielding pQM-TRMT112-mCherry and pQM-TRMT112-E2Tag, respectively. For pRS315-WBSCR22-KT/AA and pRS315-WBSCR22-D117A, the coding sequences of corresponding proteins were cut with the Acc65I, followed by Klenow treatment and XbaI digestion after which fragments were ligated into Ecl136II and XbaI sites of pRS315 vector. pQM-HA-ubi was a kind gift from Dr. Ivar Ilves (University of Tartu).

### Cell culture and DNA transfections

Human osteosarcoma cells (U2OS) (ATCC HTB-96), human cervical carcinoma cells (HeLa) (ATCC CCL-2), African green monkey kidney cells (COS-7) (ATCC CRL-1651) and human hepatocellular carcinoma cells HepG2 (ATCC HB-8065), which were obtained from the American Type Culture Collection, were grown in Iscove´s Modified Dulbecco´s Medium (IMDM) supplemented with 10% fetal calf serum (FCS), 100 U/ml penicillin and 100 μg/ml streptomycin. Cells were incubated at 37°C in 5% CO_2_ environment. To label U2OS-E2Tag-WBSCR22 cells with isotopes, the cells were grown in SILAC DMEM supplemented with 10% dialyzed fetal bovine serum, heavy arginine (0.133 mM, CNLM-539) and heavy lysine (0.266 mM; CNLM-291) (Cambridge Isotope Laboratories Inc.), for 7 days.

For DNA electroporation, 250 μl of cell suspension in IMDM was mixed with plasmid and salmon sperm carrier DNA and transfected by electroporation in 4-mm cuvettes (Thermo Fisher Scientific) using Bio-Rad GenePulser Xcell (settings 200 V, 975 μF). The cells were collected by centrifugation and resuspended in IMDM media containing 10% FCS and antibiotics and analyzed 24, 48 or 72 hours after transfection. To study the expression level and stability of WBSCR22 and TRMT112 in HeLa and U2OS cells, 500 ng of plasmid DNA was used for each transfection. 2 μg and 200 ng of plasmids were used to electroporate HepG2 and COS-7 cells, respectively.

For generation of stable cell line U2OS-E2Tag-WBSCR22, U2OS cells were transfected with dimers consisting of linearized plasmids, pQMHSP-WBSCR22 and pBabePuro. 24 hours after transfection, puromycin at final concentration 5 μg/ml was added to the media. Two weeks after transfection colonies were selected and the expression of WBSCR22 protein was analyzed by immunoblotting.

### RNA interference

The sequences of siRNAs (purchased from Sigma-Aldrich and Microsynth) used in this study are listed in S3 Table. siRNAs were purchased from Sigma-Aldrich. For siRNA knock-down, 100 μl of cell suspension (10^6^ cells) in Opti-MEM was mixed with 500 pmol of siRNA and transfected by electroporation in 4-mm cuvettes (Thermo Fisher Scientific) using Bio-Rad GenePulser Xcell (settings square wave, 1000 V, 2 pulses, pulse length 0.5 ms). The cells were suspended in IMDM medium supplemented with 10% FCS and antibiotics. 48 hours after transfection cell lysates were analyzed by western blot.

### Immunoprecipitation and western blot analysis

Cells electroporated with 200 ng (COS-7) or 1000 ng (HeLa) of plasmids pQM-CMV-E2-N/A, pQM-NTag-WB22, pQM-WBSCR22-KT/AA, pQM-WBSCR22-D117A, pQM-WBSCR22-MTD or pQM-TRMT112-E2Tag were collected 24 hours post-transfection and co-immunoprecipitation was performed as described previously [33], except for the antibody against E2Tag (clone 5E11; Icosagen) was used for immunoprecipitation. Alternatively, COS-7 cells were transfected by electroporation with plasmids pEGFP-C1 or pEGFP-WBSCR22-CTD and 24 hours after transfection, co-immunoprecipitaion using GFP-Trap_M magnetic beads (ChromoTek) was performed according to manufacturer´s protocol. The proteins of interest were detected by western blot using the mouse monoclonal antibodies anti-E2Tag antibody 5E11 (Icosagen), anti-C1QBP (sc-271201, Santa Cruz Biotechnology), anti-α-tubulin (Sigma Aldrich), anti HA-HRP (Santa Cruz Biotechnology), and rabbit polyclonal antibodies against TRMT112 (HPA040006, Sigma Aldrich), WBSCR22 (sc-135322, Santa Cruz Biotechnology) and EGFP (University of Tartu). Detection was performed using an ECL detection kit (GE Healthcare) following the manufacturer's manual.

### Proteomics analysis

The lysis and immunoprecipitation of cells was carried out in IP buffer (10 mM Tris pH 7.5, 100 mM KCl, 2 mM MgCl_2_, 1 mM DTT, 0.5% NP-40, protease inhibitors). Lysates were incubated on ice for 10 minutes and disrupted mechanically in Dounce homogenizer. After centrifugation at 13 000 rpm for 10 minutes at 4°C, the supernatant was collected and pre-cleared with protein G beads for 30 minutes. Then protein G beads conjugated with antibody against E2Tag (clone 5E11; Icosagen) were added and incubated at 4°C overnight. Beads were washed 3 times in IP buffer, and then the heavy and light samples were combined and washed another 2 times. The samples were denatured in urea buffer (7 M urea, 2 M thiourea). The proteins were reduced for 1 h at 20°C in a 1 mM dithiotreitol solution and alkylated for 1 h in 5 mM iodoacetamide in the dark. Endoproteinase LysC (Wako) was added, and the reaction mixture was incubated for 4 h at room temperature. The sample was diluted with a digestion buffer (50 mM ammonium bicarbonate in water). Trypsin (Promega) was added, and the sample was incubated overnight at room temperature. Enzyme activity was quenched by adding 1% trifluoroacetic acid, and the resulting peptides were desalted using Stage Tips. The samples were analyzed in three technical replicates with liquid chromatography tandem-mass spectrometry (LC-MS/MS) using an Agilent 1200 series nanoflow system (Agilent Technologies) connected to an LTQ Orbitrap Classic spectrometer (Thermo Electron) equipped with a nanoelectrospray ion source (Proxeon) as described previously [34]. Protein identification and quantification were performed using the MaxQuant software package (vesion 1.1.1.36). At least two peptides were required for protein identification, and two or more SILAC ratio counts were required to report a protein ratio.

### Yeast experiments

For complementation assay, pRS315 vector, pRS315-Bud23 and pRS315-WBSCR22, pRS315-WBSCR22-KT/AA, pRS315-WBSCR22-D117A and pRS315-WBSCR22-MTD were transformed into *bud23Δ* (*MAT*a *bud23*::*kanMX his3Δ1 leu2Δ0 ura3Δ0 met15Δ0*) yeast strain AJY2161 (White et al., 2008) by standard method. Yeast strains carrying pRS315 derived plasmids were cultured in synthetic complete media minus leucine supplemented with 2% glycose at 30°C. For growth dilution assays, cultures were grown overnight and diluted to final optical density at OD_600_ 0.1, from which further 10-fold serial dilutions were prepared and spotted (5 μl) onto-LEU plates. For polysome analysis, cultures were grown to optical density OD_600_ 0.3–0.5, cycloheximide was added to final concentration of 0.1 mg/ml, and the cells were collected by centrifugation. All the steps were carried out on ice. Cell pellets were washed with buffer (10 mM Tris-HCl; pH 7.5, 100 mM KCl, 10 mM MgCl_2_, 1 mM DTT, 0.1 mg/ml cycloheximide), resuspended in 300 μl of the same buffer, and broken by vortexing in the presence of glass beads. The extract was centrifuged for 10 min at 16 000 g at 4°C, and the supernatant was recovered. Six OD_260_ units were loaded onto a 10–45% sucrose gradient and centrifuged for 135 min at 36 000 rpm in a SW41 rotor (Beckman). The gradient was analyzed at OD_254_ nm.

### Live cell imaging and flow cytometry

HeLa cells were transfected by electroporation of expression plasmids pEGFP-C1, pEGFP-WBSCR22, pEGFP-WBSCR22-CTD, pEGFP-WBSCR22-MTD, pEGFP-WBSCR22-D117A, pQM-CTag-mCherry, pQM-TRMT112-mCherry. Cells were grown on 8-well format coverglass (Lab-Tek Chambered Coverglass w/cvr, Thermo Scientific) for 24 hours. Fluorescence was visualized using confocal laser scanning microscope LSM710 (Zeiss). Images were obtained with 63x lens and analyzed by ZEN2011 software. For flow cytometry, HeLa, HepG2, U2OS and COS-7 cells were transfected with plasmids expressing EGFP fusion proteins. 24, 48 and 72 hours post-transfection, cells were collected, washed with PBS and the percentage of EGFP positive cells was measured (BD Biosciences LSRII) and analyzed with FACSDiva software (BD Biosciences).

### Treatment with MG132

24 hours post-transfection, cells were treated with proteasome inhibitor MG132 (Calbiochem) at a concentration of 0 μM, 2 μM and 5 μM. 16 hours later cells were harvested and the percentage of EGFP positive cells was measured by flow cytometry (BD LSRII). For western blot analysis, cells were transfected with pQM-CMV-N/A (Icosagen) or pQM-NTag-WB22, treated with MG132 for 16 hours and analyzed by western blot. Alternatively, HeLa cells were transfected with control siRNA or siTRMT112#4, 56 hours after transfection treated with MG132 for 16 hours and analyzed by western blot.

### Ubiquitination assay

1 μg of pQM-HA-Ubi was co-transfected with pQM-NTag-WB22 or pQM-WBSCR22-D117A into HeLa cells. 24 hours post transfection cells were treated with 10 μM of MG132. After 4 hours of incubation cells were collected, washed with PBS and lysed in 100 μl lysis buffer (20 mM Tris-HCl pH 7.5, 150 mM NaCl, 1% SDS, 1 mM EDTA, 1 mM DTT, protease inhibitors) for 10 minutes at 100°C and centrifuged at 13000 rpm for 10 minutes. Protein lysate was diluted with 900 μl of dilution buffer (20 mM Tris-HCl pH 7.5, 150 mM NaCl, 0.5% NP-40, 1 mM EDTA, 1 mM DTT, protease inhibitors). For immunoprecipitation, the cell lysates were incubated with 1 μg of anti-E2Tag antibody 5E11 (Icosagen) for 2 h at 4°C, 20 μl of protein G Sepharose was added to the lysate and incubated at 4°C overnight. Beads were washed three times with 1 ml washing buffer (20 mM Tris-HCl pH 7.5, 500 mM NaCl, 0.5% NP-40, 1 mM EDTA) and eluted with 2xSDS loading buffer. The samples were analyzed by immunoblotting with anti-WBSCR22 and anti-HA antibodies.

## Supporting Information

S1 Fig Subcellular localization of WBSCR22 interacting partners TRMT112 and C1QBP. HeLa cells grown on glass coverslips were fixed and incubated with primary antibody against TRMT112 (HPA040006, Sigma Aldrich, dilution 1:50) and C1QBP (sc-271201, Santa Cruz Biotechnology, dilution 1:50), and with Alexa-568 and Alexa-488 conjugated secondary antibodies (dilution 1:1000) (Invitrogen), respectively. Fluorescence was visualized using confocal laser scanning microscope LSM710 (Zeiss). Images were obtained with 63x lens and analyzed by ZEN2011 software.
(TIF)

S2 Fig Localization of TRMT112 is determined by the WBSCR22 protein. Live cell confocal images and fluorescence intensity profile graphs of TRMT112-mCherry protein co-expressed with EGFP-WBSCR22-wt or EGFP-WBSCR22-D117A. HeLa cells were transfected with siNEG (A) or siWBSCR22#4 (B) and transfected with plasmids encoding for TRMT112-mCherry and EGFP-WBSCR22-wt or TRMT112-mCherry and EGFP-WBSCR22-D117A 48 hours after siRNA transfection. Fluorescence signal was visualized using confocal laser scanning microscope LSM710 (Zeiss). Images were obtained with 63x lens and analyzed by ZEN2011 software. (C) The expression level of WBSCR22 is response to siWBSCR22#4 treatment analyzed by western blot. Antibodies against WBSCR22 and α-tubulin were used.
(TIF)

S1 Table List of the proteins identified by SILAC analysis. (XLS)

S2 Table Oligonucleotides used in this study. (DOCX)

S3 Table siRNAs used in this study. (DOCX)
