# Supplementary figures and images for: The Stability of Ribosome Biogenesis Factor WBSCR22 Is Regulated by Interaction with TRMT112 via Ubiquitin-Proteasome Pathway
Source: PLoS One. 2015 Jul 27;10(7):e0133841. doi: 10.1371/journal.pone.0133841 (PMC4516353; doi:10.1371/journal.pone.0133841)

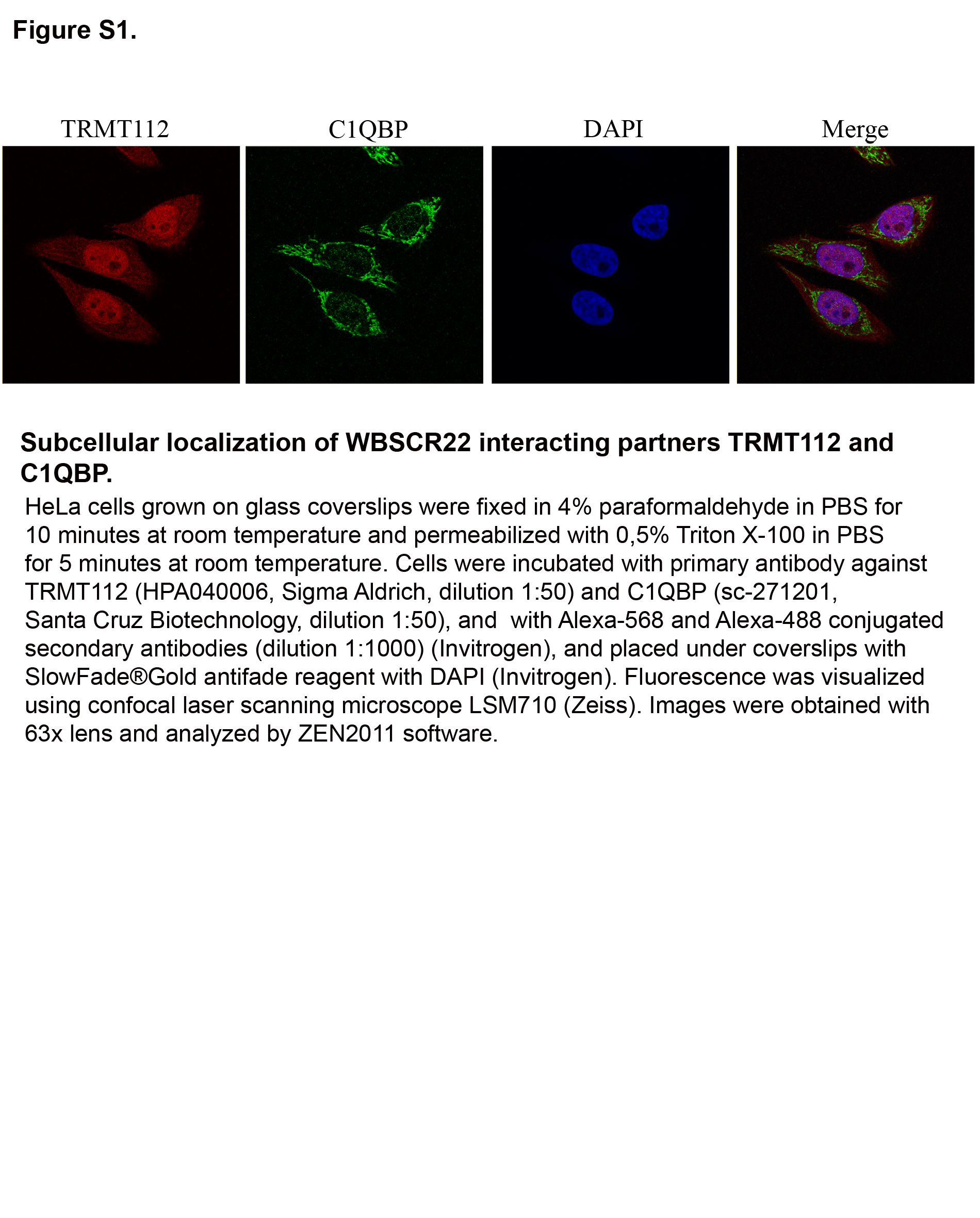

Supplement: S1 Fig — HeLa cells grown on glass coverslips were fixed and incubated with primary antibody against TRMT112 (HPA040006, Sigma Aldrich, dilution 1:50) and C1QBP (sc-271201, Santa Cruz Biotechnology, dilution 1:50), and with Alexa-568 and Alexa-488 conjugated secondary antibodies (dilution 1:1000) (Invitrogen), respectively. Fluorescence was visualized using confocal laser scanning microscope LSM710 (Zeiss). Images were obtained with 63x lens and analyzed by ZEN2011 software. (TIF) [file pone.0133841.s001.tif]

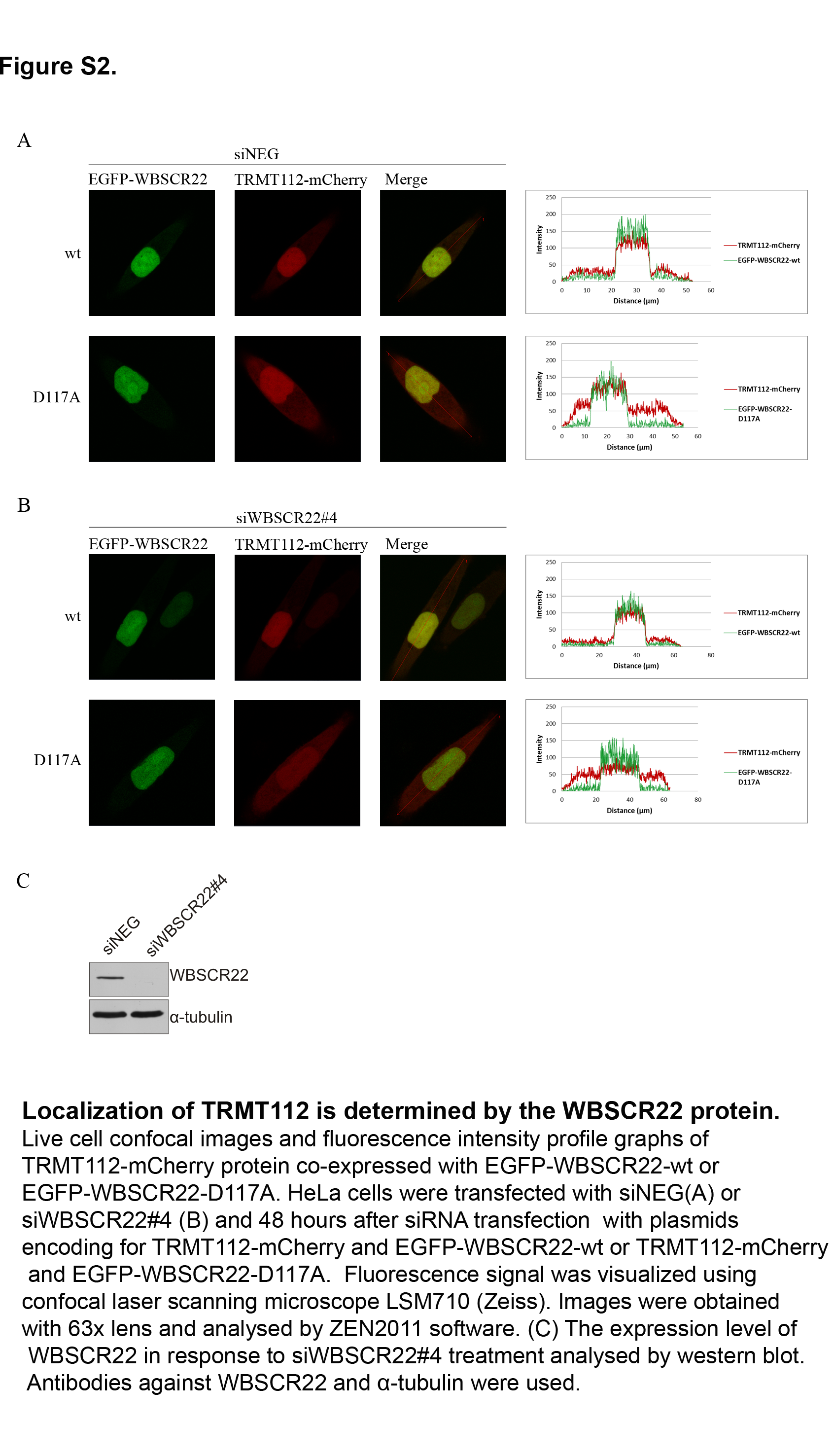

Supplement: S2 Fig — Live cell confocal images and fluorescence intensity profile graphs of TRMT112-mCherry protein co-expressed with EGFP-WBSCR22-wt or EGFP-WBSCR22-D117A. HeLa cells were transfected with siNEG (A) or siWBSCR22#4 (B) and transfected with plasmids encoding for TRMT112-mCherry and EGFP-WBSCR22-wt or TRMT112-mCherry and EGFP-WBSCR22-D117A 48 hours after siRNA transfection. Fluorescence signal was visualized using confocal laser scanning microscope LSM710 (Zeiss). Images were obtained with 63x lens and analyzed by ZEN2011 software. (C) The expression level of WBSCR22 is response to siWBSCR22#4 treatment analyzed by western blot. Antibodies against WBSCR22 and α-tubulin were used. (TIF) [file pone.0133841.s002.tif]
