# Supplementary material for: The Stability of Ribosome Biogenesis Factor WBSCR22 Is Regulated by Interaction with TRMT112 via Ubiquitin-Proteasome Pathway
Source: PLoS One. 2015 Jul 27;10(7):e0133841. doi: 10.1371/journal.pone.0133841 (PMC4516353; doi:10.1371/journal.pone.0133841)
Supplement: S2 Table — (DOCX) [file pone.0133841.s004.docx]

| **TABLE S2.** Oligonucleotides used in this study | |
| --- | --- |
| Name | Sequence |
| CherC-F | CGGGGTACCATGGTGAGCAAGGGC |
| CherC-R | GGAAGATCTTCACTTGTACAGCTCGTC |
| pCG_As | GCCAGAAGTCAGATGCTCAAG |
| pCGseq2 | GGGACCGATCCAGCCTCC |
| TRMTBgl-F | CAAGATCTCTTATGAAACTGCTTACC |
| TRMT-R | TAGGTACCACTCTCAGTTTCCTCTTC |
| WB_D117A_F | ACATTTG**C**TGGTTGCATC |
| WB_K112T115AA_F | CCATTCGCGCCAGGCGCATTTGATG |
| WB22_aa244 | ACCCAAGCTTCAATGGTGAGGAAGAGTC |
| WB22-BglF | CAAGATCTTCTATGGCGTCCCGCGGCC |
| WB22del2R | CCATAGATCTTTACCCAGAAAACAAGCAGAG |
| WB22-KpnR2 | GGGGTACCCCACTTAGAAGCGGGGCTTGCG |
