# Supplementary material for: The Stability of Ribosome Biogenesis Factor WBSCR22 Is Regulated by Interaction with TRMT112 via Ubiquitin-Proteasome Pathway
Source: PLoS One. 2015 Jul 27;10(7):e0133841. doi: 10.1371/journal.pone.0133841 (PMC4516353; doi:10.1371/journal.pone.0133841)
Supplement: S3 Table — (DOCX) [file pone.0133841.s005.docx]

| **TABLE S3.** Sequences of siRNAs used in this study | |
| --- | --- |
| Name | Sequence |
| negative siRNA | 5´-CACCGACGGAGACAAAGCUUU-3´ |
| siC1QBP | 5´-CACCGACGGAGACAAAGCUUU-3´ |
| siTRMT112#1 | 5´-AACUUCGUGGCGCGUAUGAUA-3´ |
| siTRMT112#2 | 5´-AAUCUGCUGAGCUCGCAUGUG-3´ |
| siTRMT112#3 | 5´-UGUUGAUCUAUACCCUGUU-3´ |
| siTRMT112#4 | 5´-CGAAUUCUGCCGUGUGUAU-3´ |
| siTRMT112#5 | 5´-CAAUGACACCAAACACAGU-3´ |
| siWBSCR22  siWBSCR22#4 | 5´-CGAGCAUUGGAGCUUCUUUAU-3´  5´-CUGACAAAGUAGUAUUUUA-3´ |
